# VCAM1-α4β1 integrin interaction mediates interstitial tissue reconstruction in 3-D re-aggregate culture of dissociated prepubertal mouse testicular cells

**DOI:** 10.1038/s41598-021-97729-y

**Published:** 2021-09-15

**Authors:** Kazuko Abe, Shigeyuki Kon, Hiroki Kameyama, JiDong Zhang, Ken-ichirou Morohashi, Kenji Shimamura, Shin-ichi Abe

**Affiliations:** 1 Faculty of Health Science, Kumamoto Health Science University, 325 Izumi-machi, Kita-ku, Kumamoto, 861-5598 Japan; 2 Department of Molecular Immunology, Faculty of Pharmaceutical Sciences, Fukuyama University, Fukuyama, Hiroshima Japan; 3 School of Basic Medical Sciences, ZunYi Medical University, Zunyi, Guizhou Province China; 4 Department of Molecular Biology, Graduate School of Medical Sciences, Kyushu University, Fukuoka, Japan; 5 Institute of Molecular Embryology and Genetics, Kumamoto University, Kumamoto, Japan

**Keywords:** Morphogenesis, Organogenesis, Endocrinology

## Abstract

Roles of interstitial tissue in morphogenesis of testicular structures remain less well understood. To analyze the roles of CD34^+^ cells in the reconstruction of interstitial tissue containing Leydig cells (LCs), and testicular structures, we used 3D-reaggregate culture of dissociated testicular cells from prepubertal mouse. After a week of culture, adult Leydig cells (ALCs) were preferentially incorporated within CD34^+^ cell-aggregates, but fetal LCs (FLCs) were not. Immunofluorescence studies showed that integrins α4, α9 and β1, and VCAM1, one of the ligands for integrins α4β1 and α9β1, are expressed mainly in CD34^+^ cells and ALCs, but not in FLCs. Addition of function-blocking antibodies against each integrin and VCAM1 to the culture disturbed the reconstruction of testicular structures. Antibodies against α4 and β1 integrins and VCAM1 robustly inhibited cell-to-cell adhesion between testicular cells and between CD34^+^ cells. Cell-adhesion assays indicated that CD34^+^ cells adhere to VCAM1 through the interaction with α4β1 integrin. Live cell imaging showed that CD34^+^ cells adhered around ALC-aggregates. CD34^+^ cells on the dish moved toward the aggregates, extending filopodia, and entered into them, which was disturbed by VCAM1 antibody. These results indicate that VCAM1-α4β1 integrin interaction plays pivotal roles in formation of testicular interstitial tissues in vitro and also in vivo.

## Introduction

Vertebrate testis basically comprises seminiferous tubules and interstitial tissues. The former consist of Sertoli cells (SCs) and germ cells, and SCs form the basic structure of seminiferous tubules, while interstitial tissues contain Leydig cells (LCs), vascular cells, immune cells such as macrophages, and mesenchymal cells. Roles of interstitial cells in testicular morphogenesis and functions in spermatogenesis have been reported by some investigators^1,2^. However, how the interstitial tissues are formed and maintained is less well understood. Especially the roles of CD34^+^ cells which have been called as mesenchymal cells, stromal cells or telocytes are not well clarified yet. CD34^+^ cells are distributed in many organs of mammals and involved in morphogenesis and regeneration of various tissues such as heart, skin, etc.^3–5^. The mechanisms underlying testicular morphogenesis, and cell–cell interactions and functions of various factors involved, have long been studied embryologically and genetically^6–8^, yet many issues still remain to be resolved. A powerful alternative approach to elucidate the mechanisms by which cell–cell interactions and molecular signals operate to drive the morphogenesis is to establish three-dimensional (3D) culture models in which dissociated cells reconstruct the original structures, as studied in kidney, lung, etc.^9,10^. We have recently succeeded in the reconstruction of seminiferous tubule-like structures in vitro using a 3-D culture of dissociated testicular cells from prepubertal mouse^11,12^. Dissociated testicular cells cultured inside a collagen matrix form within a week seminiferous tubule-like structures with lumen within which germ cells reside, and peritubular myoid cells (PMCs) attach around the SC-re-aggregates; outside the seminiferous tubule-like structures CD34^+^ cells form interstitial tissues within which LCs are localized. We have also shown the important roles of the interstitial tissues of the testis: testicular cells deprived of interstitial cells form dysmorphic structures, while re-addition of the interstitial cells restored tubule-like structures^12^.

LCs, a major component of testicular interstitium, play indispensable roles in spermatogenesis by secreting androgens under the stimulus of luteinizing hormone (LH). It is well known that two types of LCs, namely fetal LCs (FLCs) and adult LCs (ALCs), appear during mammalian testis development^13^. The former develop during the fetal stage, decline after birth, but persist throughout adulthood, while the latter appear after birth and develop until adulthood. They show some different features in morphology, function in steroid synthesis, as well as gene expression patterns^13–17^. FLCs form clusters in the interstitium of the testis in late fetal stages. ALCs arise from stem cells at places just outside seminiferous tubules and differentiate into mature ALCs which eventually reside as clusters in broad spaces in the interstitium in adults. But it is unknown how ALC stem cells differentiate and move to their final location and form clusters as mature ALCs.

Integrins are transmembrane receptors consisting of two heterodimeric subunits, the α-subunit and β-subunit, and constitute a family with 24 different combinations of the two subunits^18^. Integrins play pivotal roles in regulation of diverse cellular functions such as adhesion to extracellular matrix (ECM) proteins, migration, proliferation, apoptosis and differentiation^19,20^. α9 and α4 integrins belong to the same subfamily due to their homologous sequences and form heterodimers with β1 integrin^18–21^. α9β1 integrin is expressed in various tissues, and plays important roles in the development of autoimmune diseases such as rheumatoid arthritis and multiple sclerosis^21^. α4β1 integrin is also expressed in many tissues such as in all hematopoietic lineage precursors and T and B lymphocytes, and plays important roles in the homing of hematopoietic stem cells to the bone marrow and in the recruitment of leukocytes in inflammation^22^. Both α9β1 and α4β1 integrins bind to various ligands including some components of ECM such as osteopontin, fibronectin-EIIIA, and also VCAM1^18–21^. VCAM1 is a transmembrane glycoprotein predominantly expressed in endothelial cells of tissues such as lymph nodes and bone marrow, where it plays important roles in regulating leukocyte homing via its adhesion function^22^. VCAM1 is one of the genes expressed in ALCs but not in FLCs^14^.

In the current study, to address the mechanism underlying reconstruction of ordered seminiferous tubule-like and interstitial tissue-like structures, in the latter of which CD34^+^ cells re-assemble to form tight re-aggregates, and LCs become located within the re-aggregates of CD34^+^ cells, we first examined the behavior of CD34^+^ cells and LCs during 3-D re-aggregate culture of testicular cells. Second, we scrutinized the expression of integrins α4, α9, and β1, and VCAM1 in prepubertal testes. Since we found the expression of those integrins and VCAM1 mainly in CD34^+^ cells and ALCs, third, we examined the effects of function-blocking antibodies against each integrin and VCAM1 on the reconstruction of seminiferous tubule-like and interstitial tissue-like structures in re-aggregate cultures of testicular cells. Fourth, we assessed the effects of those antibodies on cell–cell adhesion of testicular cells and of purified CD34^+^ cells, and adhesion of CD34^+^ cells to VCAM1. Fifth, we observed direct interactions between CD34^+^ cells and LCs by live cell imaging. The results indicate that VCAM1-α4β1 integrin interaction plays pivotal roles in reconstruction of testicular interstitial tissues in vitro, and will give a clue to address the question how ALCs move to their final location and form clusters as mature ALCs.

## Results

### Behavior of CD34^+^ cells and LCs during re-aggregate culture

**Figure 1:**
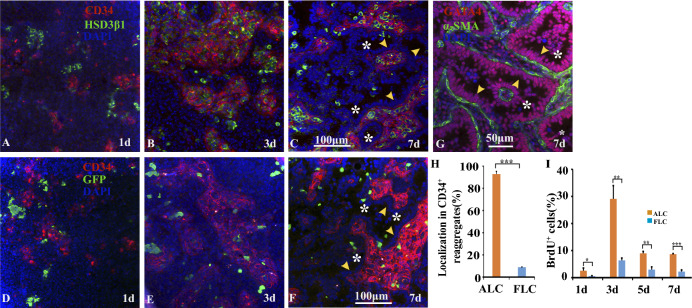
FLCs and ALCs showed different properties in re-aggregate culture of testicular cells. (**A**–**F**) Immunofluorescence of CD34, HSD3β1 (**A**–**C**)/GFP (**D**–**F**) and DAPI on day 1 (**A**,**D**), day 3 (**B**,**E**) and day 7 (**C**,**F**). (**G**) shows immunofluorescence of GATA4 (a marker of SCs and LCs), α-SMA and DAPI on day 7. Arrowheads and * show a single layer of SCs and lumen, respectively, in seminiferous tubules formed on day 7. Negative controls for the antibodies used in (**A**-**G**) were shown in Supplementary Fig. S1. (**H**) Percentages of ALCs and FLCs which were localized within CD34^+^ cell-aggregates among all the ALCs and FLCs, respectively, in sections on day 7 were shown. The former was obtained as described in Supplementary Methods. Total numbers of HSD3β1-positive cells and GFP-positive cells counted was 1916 and 538, respectively, in 3 experiments. (**I**) Proliferative activity of ALCs and FLCs during culture. The percentage of BrdU^+^ALCs was obtained as described in Supplementary Methods. Total numbers of HSD3β1-positive cells counted for each day in 3 experiments were 1492 (1d), 2107 (3d), 1507 (5d), and 1774 (7d), while those of GFP-positive cells counted were 534 (1d), 620 (3d), 527 (5d), and 671 (7d). Student’s *t*-test was performed for comparison between ALCs and FLCs in (**H**) and (**I**). *** < 0.001, ** < 0.01, * < 0.05.

In order to discern FLCs and ALCs, we used transgenic mice (*mFLE-EGFP*) in which GFP is expressed only in FLCs^23^. Thus, cells which expressed GFP were identified as FLCs, while cells which expressed HSD3β1 included both FLCs and ALCs. On day 1, HSD3β1-positive cells (ALCs and FLCs) were present as single cells or aggregates (Fig. 1A), and some single cells were intermingled with CD34^+^ cells, while the rest were located apart from the CD34^+^ cell-aggregates. Most of the HSD3β1-positive cell-aggregates were situated apart from the CD34^+^ cell-aggregates. On the other hand, FLCs were distributed as single cells or aggregates consisting of several cells, in close to, but clearly apart from CD34^+^ cell-aggregates in which the cells seemed not to adhere firmly with each other (Fig. 1D). On day 3, CD34^+^ cells formed tightly packed aggregates (Fig. 1B,E). Most of the HSD3β1-positive cells were within the CD34^+^ cell-aggregates (Fig. 1B). In contrast, a few GFP-positive cells were incorporated into the CD34^+^ cell-aggregates, whereas most of the GFP-positive cells were not (Fig. 1E). On day 7, most of the HSD3β1-positive cells were within the CD34^+^ cell-aggregates, where GATA4-positive Sertoli cells (arrowheads in Fig. 1G) formed seminiferous tubule-like structures with lumen (* in Fig. 1C,F,G)^11^, while most of the FLCs were still localized outside the CD34^+^ cell-aggregates (Fig. 1F). The percentage of the GFP-positive FLCs localized within CD34^+^ cell-aggregates among all the FLCs present on day 7 was 8.9 ± 0.2% (Fig. 1H). On the other hand, the percentage of the ALCs localized within CD34^+^ cell-aggregates among all the ALCs present on day 7 was 92.8 ± 2.4% (calculated as in Supplementary Methods) (Fig. 1H). These results indicate that ALCs were preferentially incorporated within CD34^+^ cell-aggregates, but FLCs were not. During the culture, the number of HSD3β1-positive cells seemed to increase. Thus, we assessed the proliferative activity of HSD3β1-positive and GFP-positive cells by BrdU incorporation (Fig. 1I). The proliferative activities of ALCs (calculated as in Supplementary Methods) and FLCs on day 1 were as low as 2.6 ± 1.0% and 0.6 ± 0.1%, respectively, but increased to the highest level on day 3: 29.1 ± 4.9% for ALCs and 6.4 ± 0.8% for FLCs. These results indicate that the proliferative activity of ALCs was about 4.5 times higher than that of FLCs. The proliferative activities of ALCs and FLCs decreased on days 5 and 7, but those of ALCs were still higher than those of FLCs by 3–4 times.

### Expressions of integrins α4, α9 and β1, and VCAM1

**Figure 2:**
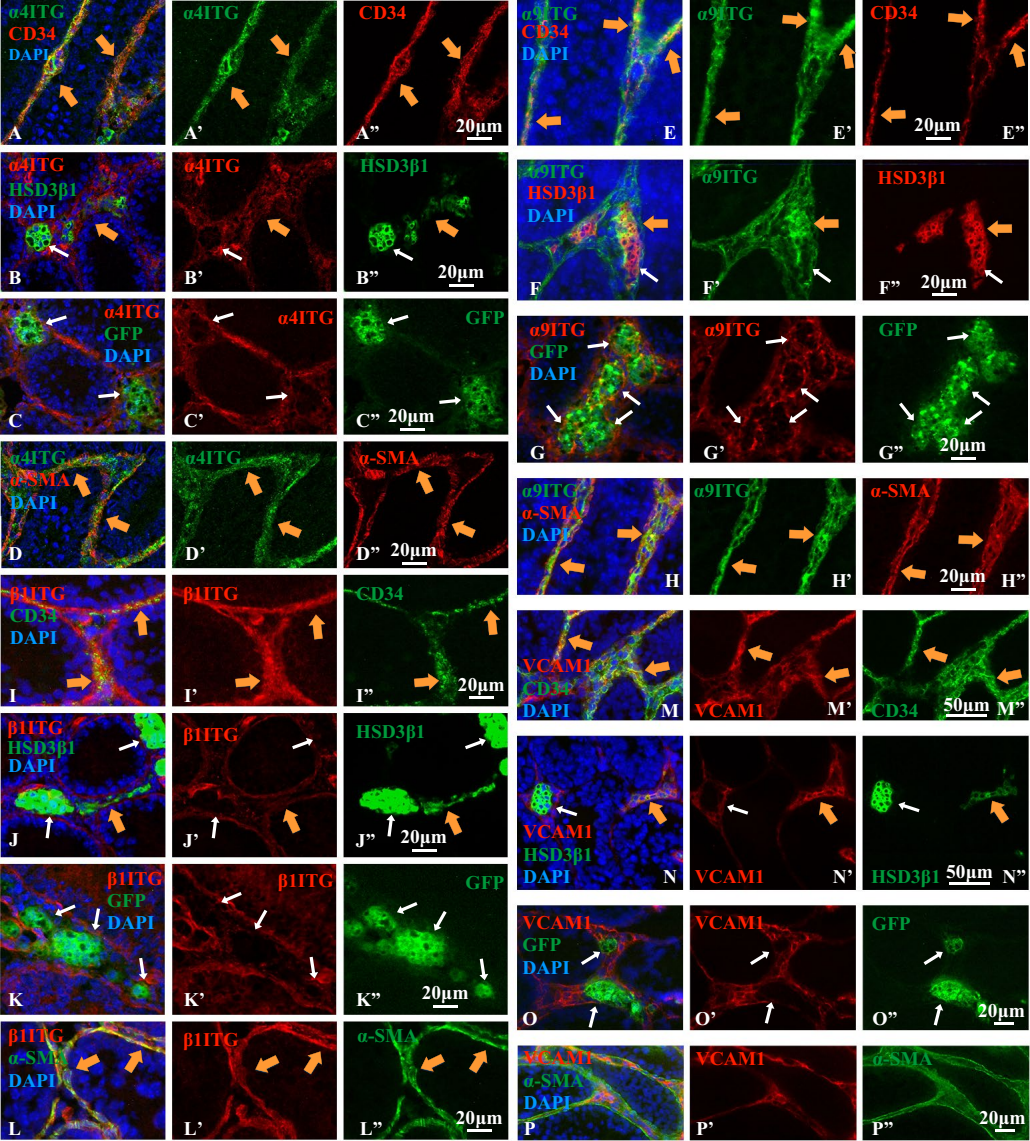
Immunofluorescence staining showed that integrins α4, α9 and β1 are expressed in CD34^+^ cells, ALCs and PMCs, but not in FLCs in 14-dpp testes. VCAM1 is also expressed in CD34^+^ cells and ALCs, but not in FLCs or PMCs. For co-staining with antibodies against GFP and those against integrin α4, α9 or β1, testes from *mFLE-EGFP* transgenic mice were used. (**A**–**D″**) Double staining for α4 integrin (ITG) (**A**,**A’**,**B**,**B’**,**C**,**C’**,**D**,**D’**), and CD34 (**A**,**A″**), HSD3β1 (**B**,**B″**), GFP (**C**,**C″**), or α-SMA (**D**,**D″**). (**E**–**H″**) Double staining for α9 ITG (**E**,**E’**,**F**,**F’**,**G**,**G’**,**H**,**H’**), and CD34 (**E**,**E″**), HSD3β1 (**F**,**F″**), GFP (**G**,**G″**), or α-SMA (**H**,**H″**). (**I**–**L″**) Double staining for β1 ITG (**I**,**I’**,**J**,**J’**,**K**,**K’**,**L**,**L’**) and CD34 (**I**,**I″**), HSD3β1 (**J**,**J″**), GFP (**K**,**K″**) or α-SMA (**L**,**L″**). (**M**–**P″**) Double staining for VCAM1 (**M**,**M’**,**N**,**N’**,**O**,**O’**,**P**,**P’**), and CD34 (**M**,**M″**), HSD3β1 (**N**, **N″**), GFP (**O**,**O″**) or α-SMA (**P**,**P″**). Cells were also stained with DAPI in (**A**–**P**). Thick orange arrows indicate regions where expressions of 2 antigens were overlapped among 3 photographs which were labeled with the same letter (e.g. **A**, **A’**, and **A″**). White thin arrows show regions where expression of HSD3β1 or GFP was not overlapped with that of α4, α9, and β1ITGs and VCAM1 (e.g. **C**, **C’**, and **C″**). All of the negative controls for the antibodies used were shown in Supplementary Fig. S1.

Since we found expressions of integrins α4, α9 and β1, and VCAM1 in the interstitial tissue based on the transcriptomes^24^, we investigated by immunofluorescence study in what cell types integrins α4, α9 and β1, and VCAM1 are expressed, namely CD34^+^ cells, ALCs, FLCs, or PMCs. Integrins α4 (Fig. 2A–A″), α9 (Fig. 2E–E″), β1 (Fig. 2I–I″) and VCAM1(Fig. 2M–M″) were clearly expressed in most, if not all, of the CD34^+^ cells, when 100–150 cells were examined in each of 3 sections from 3 wild-type testes. When co-expressions of HSD3β1 and α9ITG (Fig. 2F–F″), or VCAM1(Fig. 2N–N″) were examined, 64.4 ± 0.57% and 65.7 ± 0.47% of the HSD3β1-positive cells showed overlapped expression with integrinα9 and VCAM1, respectively, whereas the rest of them did not. Taking into account that 66.0 ± 1.4% of the HSD3β1-positive cells are ALCs when examined in *mFLE-EGFP* testes, most of the ALCs were considered to express α9ITG and VCAM1. Some of the HSD3β1-positive cells were found to co-express integrin α4 (Fig. 2B–B″) and β1 (Fig. 2J–J″), though quantification of the percentage of the overlapped cells was difficult due to weak expressions. On the other hand, very few GFP-positive cells expressed integrin α4 (Fig. 2C–C″), α9 (Fig. 2G–G″), β1 (Fig. 2K–K″), or VCAM1 (Fig. 2O–O″). These results indicate that very few FLCs express integrins α4, α9, β1,and VCAM1. Integrins α4 and α9 seemed to overlap weakly with most of the α–SMA-positive PMCs (Fig. 2D–D″,H–H″). Integrin β1 was expressed in most of the PMCs and some seminiferous tubules (Fig. 2L–L″). VCAM1 was expressed in very few PMCs (Fig. 2P–P″).

### Function-blocking antibodies against integrins α4, α9, and β1, and VCAM1 disturbed the normal formation of interstitial tissue-like as well as seminiferous-tubule-like structures

**Figure 3:**
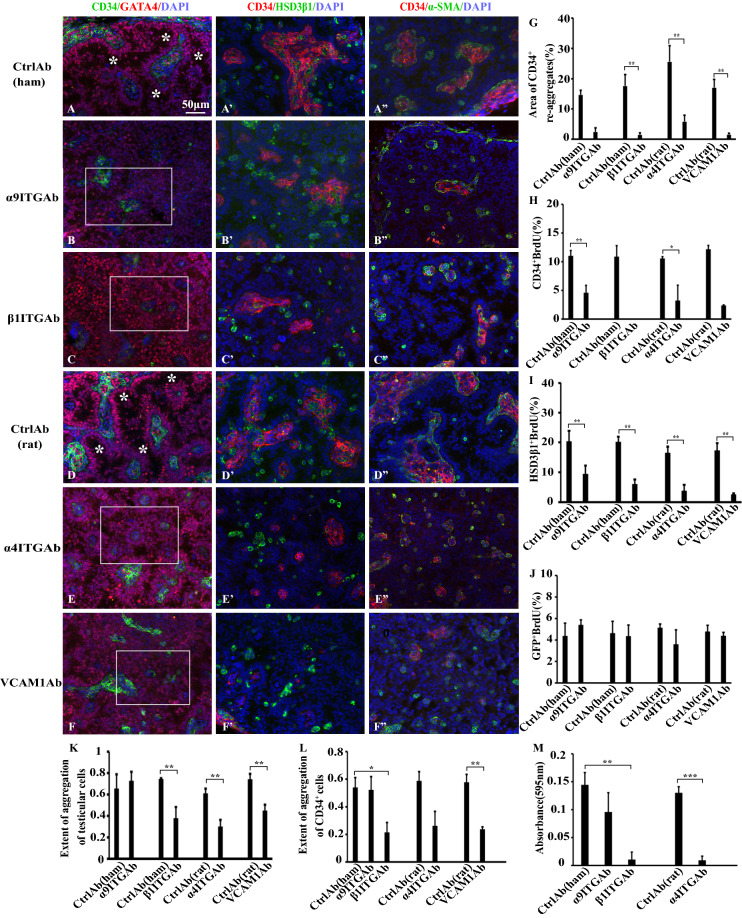
Addition of function-blocking antibodies against α9, β1 and α4 integrins, and VCAM1 disturbed the reconstruction of seminiferous tubule-like and interstitial tissue-like structures in re-aggregate cultures. (**A**–**F″**) Sections of the re-aggregates cultured for 7 days in the presence of control antibodies {Ctrl Ab (ham) (**A**–**A″**) or Ctrl Ab (rat) (**D**–**D″**)} or antibodies against α9 integrin (ITG) (**B**–**B″**), β1 ITG (**C**–**C″**), α4 ITG (**E**–**E″**), or VCAM1 (**F**–**F″**) were immuno-stained with antibodies against CD34 and GATA4 (**A**–**F**), HSD3β1 (**A’**–**F’**), or α-SMA (**A″**–**F″**). All sections were also stained with DAPI. All of the GATA4-positive cells are Sertoli cells, except those in the interstitial tissue (within CD34^+^ cell-aggregates) in (**A**) and (**D**) are very probably LCs. All of the negative controls for the antibodies used in (**A**–**F″**) were shown in Supplementary Fig. S1. * in (**A**) and (**D**) shows lumen within seminiferous tubule-like structures formed. White rectangles in (**B**), (**C**), (**E**) and (**F**) show representative areas displaying multi-cell layers of Sertoli cells around small and spherical interstitial tissue-like structures. (**G**) Percentage of areas occupied by CD34^+^ cell-aggregates among total areas of the sections of re-aggregates cultured in the presence of each antibody shown. (**H**–**J**) Percentage of BrdU-positive CD34^+^ cells (**H**), HSD3β1^+^ cells (ALCs and FLCs) (**I**), and GFP-positive cells (FLCs) (**J**) in the presence of each antibody shown. (**H**) 1.1–2.3 × 10^3^ CD34 + cells were counted in the presence of each antibody except β1ITG Ab (27 cells) and α4ITG Ab (157 cells) for 3 experiments. (**I**) 2.0–9.6 × 10^2^ HSD3β^+^ cells, and (**J**) 5.1–9.0 × 10^2^ GFP-positive cells were counted in the presence of each antibody for 3 experiments. (**K**,**L**) Extent of cell–cell adhesion of dissociated testicular cells (**K**) and of purified CD34^+^ cells (**L**) in the presence of each antibody shown. M) Cell-VCAM1 adhesion of purified CD34^+^ cells in the presence of each antibody shown. Summary of the statistical analysis was shown as Supplementary Table S2. ***p < 0.001, **p < 0.01, *p < 0.05.

To investigate the functions of integrins α4, α9 and β1, and VCAM1 in the reconstruction of interstitial-like structures as well as seminiferous-tubule like structures, function-blocking antibodies against each integrin and VCAM1 were applied during re-aggregate culture. Cultures treated with control antibodies (hamster and rat) showed, after 1 week of culture, formation of ordered structures of interstitial tissue-like and seminiferous tubule-like tissues; the former consisted of CD34^+^ cells and LCs, surrounded by a layer of PMCs (Fig. 3A–A″,D–D″); the latter consisted of mostly one layer of Sertoli cells lining along a layer of PMCs, and lumen (* in Fig. 3A,D; see also Fig. 1C,F,G). Many LCs were within the CD34^+^ cell-re-aggregates (Fig. 3A’,D’). On the other hand, addition of antibodies against integrin α4, α9 or β1, or VCAM1 caused formation of irregular structures; Sertoli cells attached around interstitial tissues mostly as a multi-cell layer, forming a rosette-like pattern (Fig. 3B,C,E,F). Areas of interstitial tissues comprising CD34^+^ cells as well as those of lumen within seminiferous-tubules became smaller than those in controls. Indeed, the former was 5–10 times smaller than that of controls (Fig. 3G). A greater number of LCs were outside than inside of the interstitial tissues (compare Fig. 3A’,D’ with Fig. 3B’,C’,E’,F’). The smaller area of the interstitial tissues and number of LCs could have been due to decreased proliferative activities of CD34^+^ cells and LCs caused by the antibodies. Indeed, the proliferative activities of CD34^+^ cells on day 3 were downregulated by antibodies against integrin α4, α9 and β1, and VCAM1 (Fig. 3H). The combined proliferative activities of ALCs and FLCs were also suppressed by these antibodies (Fig. 3I), whereas the proliferative activity of FLCs was not (Fig. 3J). In addition, many PMCs in the samples treated with these antibodies were present as single round cells (Fig. 3B″,C″,E″,F″) compared with those in controls (Fig. 3A″,D″). Overall, the effects of α4 and β1 integrin antibodies and VCAM1 antibody were more robust than that of α9 integrin antibody. These results indicate that integrins α4β1 and α9β1, and VCAM1 play pivotal roles in reconstruction of seminiferous tubule-like and interstitial-like structure.

### Antibodies against α4 integrin, β1 integrin and VCAM1 suppressed cell-to-cell adhesions of testicular cells and of CD34^+^ cells, and cell adhesion of CD34^+^ cells to recombinant VCAM1

Aberrant interstitial tissue-like and seminiferous tubule-like structures that were formed in re-aggregate cultures upon the addition of antibodies against integrins α4, α9 and β1, and VCAM1 could be partly due to the disturbances of cell–cell adhesion between testicular cells. To check this possibility, we performed cell–cell aggregation assays of total testicular cells (Fig. 3K). When dissociated testicular cells were treated with α9 integrin antibody, the extent of aggregation was not so different from that treated with control hamster antibody. Addition of antibodies against β1 integrin, α4 integrin and VCAM1 to the cells significantly decreased the extent of aggregation, when compared with each control antibody (Fig. 3K). Next, we assessed the effect of those antibodies on the aggregation of purified CD34^+^ cells, because these cells are a major population of the interstitial tissue (Fig. 3L). α9 integrin antibody had no inhibitory effect on the aggregation. Treatment of the cells with antibodies against β1 integrin and VCAM1 significantly suppressed the extent of aggregation to the level lower than the half of each control. α4 integrin antibody also suppressed the aggregation to about the half level of the control, though not significant. These results indicate that cell–cell adhesion of CD34^+^ cells is mediated by α4β1 integrin and VCAM1.

Then we investigated whether CD34^+^ cells can bind to recombinant VCAM1 by performing cell adhesion assays (Fig. 3M). Treatment of the cells with β1 integrin antibody or α4 integrin antibody robustly suppressed the adhesion to VCAM1, compared with hamster IgG-treated or rat IgG-treated cells, respectively. However, α9 integrin antibody treatment had no significant effect. These results show that CD34^+^ cells are able to bind to VCAM1 through α4β1 integrin, indicating that CD34^+^ cells adhere to each other through the interaction between α4β1 integrin and VCAM1.

### Live imaging shows how CD34^+^ cells interact with LCs

In order to examine how CD34^+^ cells interact with LCs, we observed their behavior by live cell imaging when both cell-types were mixed and cultured for 1–3 days. For the study, we utilized another *EGFP* transgenic mouse, *Ad4BP-BAC-EGFP*, in which both FLCs and ALCs are labeled by EGFP^25^. Because both LCs and CD34^+^ cells derived from *Ad4BP-BAC-EGFP* mouse testes were GFP-positive (Supplementary Fig. S2), we used GFP-positive LCs isolated from the mice and GFP-negative CD34^+^ cells purified from wild-type or *mFLE-EGFP* transgenic mouse testes (Supplementary Fig. S2). The percentage of GFP-positive ALCs and FLCs among the total testicular cells which were isolated from *Ad4BP-BAC-EGFP* mouse (15-dpp) and applied to the sorter was 15–20%, while the GFP-positive FLCs among the total testicular cells which were isolated from *mFLE-EGFP* transgenic mouse at the same stage and applied to the sorter was lower than 1.0%. Thus GFP-positive LCs we observed were considered to be mostly ALCs.

**Figure 4:**
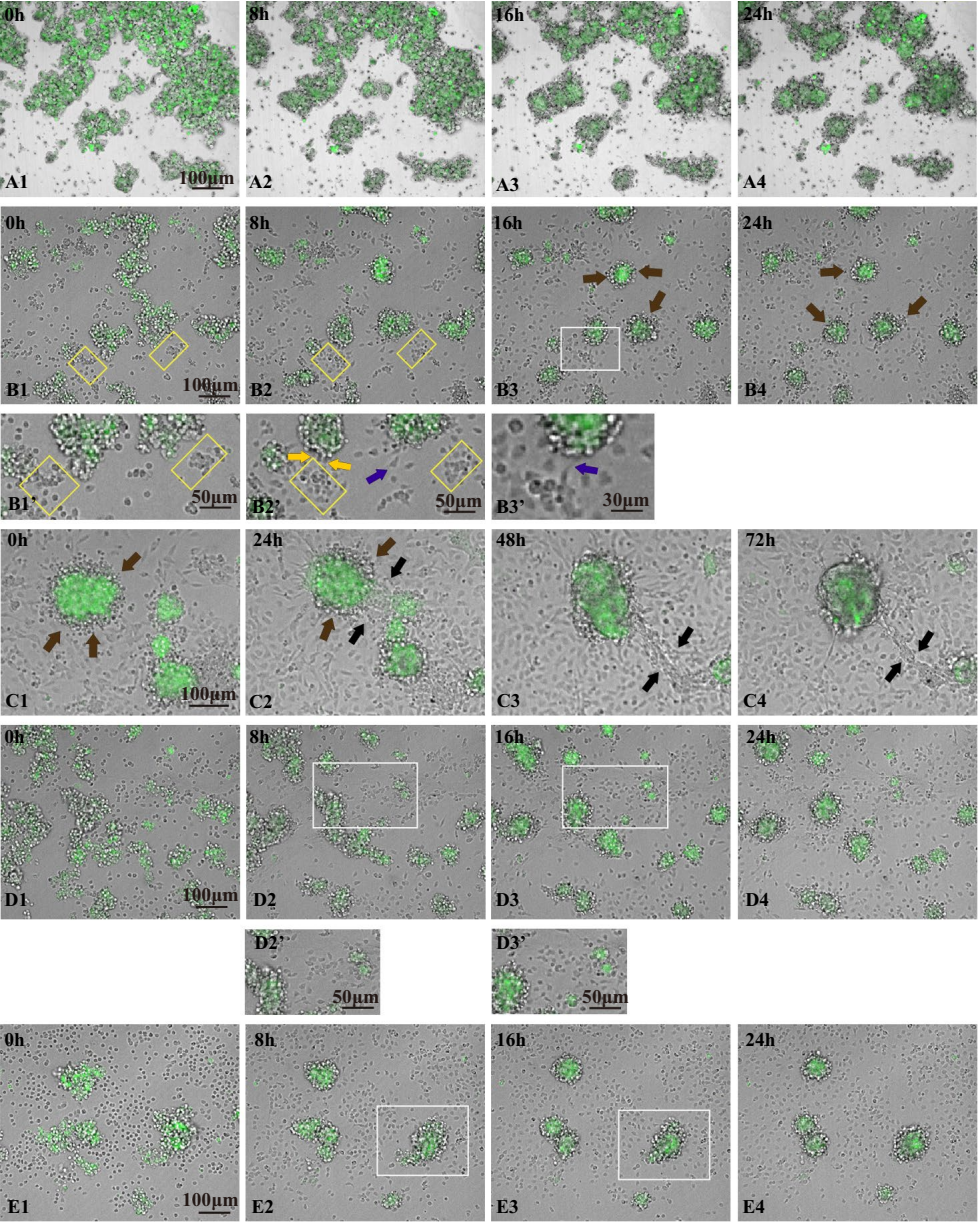
Live cell imaging shows interaction of CD34^+^ cells and ALCs. Merged figures of GFP and bright field images are shown. All photographs shown here are slice snapshots except (**A1**–**A4**) which are snapshots of MIPs. (**A1**–**A4**) Behavior of purified LCs only (GFP-positive, mostly ALCs) recorded by time-lapse video camera during 24 h. (**B1**–**B4**) Behavior of LC-CD34^+^ cell-aggregates and CD34^+^ cells which were spread around the aggregates and adhered to the dish. Areas surrounded by yellow boxes in (**B1**) and (**B2**) show regions where CD34^+^ cells that adhered to each other gradually spread. Enlarged figures around the yellow boxed areas in (**B1**) and (**B2**) are also shown in (**B1’**) and (**B2’**). Orange arrows in (**B2’**) show CD34^+^ cells which are just plunging into LC-CD34^+^ cell-aggregates. A purple arrow in (**B2’**) shows a CD34^+^ cell which was going toward a LC-CD34^+^ cell-aggregate. Brown arrows in (**B3**), (**B4**), (**C1**) and (**C2**) show round CD34^+^ cells that attached around LC-aggregates. (**B3’**) is the enlarged figure of the white-boxed area in (**B3**), and a purple arrow shows an example of a CD34^+^ cell which extended filopodia and was just touching the LC-CD34^+^ cell-aggregate. (**C1**–**C4**) are an example showing that some LC-CD34^+^ cell-aggregates were connected by cytoplasmic processes extending from the 2 aggregates (black arrows in **C2**–**C4**) and coalesced into one aggregate (**C3**,**C4**). In this case, starting time was 17.5 h following inoculation. (**D1**–**D4**) show an example of the behavior of CD34^+^ cells treated with VCAM1 antibody. White boxed areas (**D2**,**D3**) and their enlarged figures (**D2’**,**D3’**) show the typical behavior of CD34^+^ cells. (**E1**–**E4**) show an example of the behavior of CD34^+^ cells treated with antibodies against α4, α9 and β1 integrins.

When GFP-positive LCs only were treated with control antibodies, and incubated for 30 min at 34 °C, they adhered to each other and formed re-aggregates (Fig. 4A1). When the LCs were observed continually, the LC-aggregates became roundish within a day, but they gradually ceased to fluoresce with GFP, indicating that they were going to die (Fig. 4A2–A4, Supplementary Video S1). When LCs and CD34^+^ cells, both of which were treated with control antibodies, were mixed, the two cell types adhered to each other (Fig. 4B1). These aggregates gradually became roundish, but more condensed than the aggregates of LCs only (Fig. 4B2–B4, Supplementary Video S2). These LC-aggregates with CD34^+^ cells survived longer than LC-only aggregates, indicating the contributions of CD34^+^ cells to promoting the survival of LCs (Fig. 4B2–B4,C1–C4). At the same time GFP-positive LCs came to be localized inside, with CD34^+^ cells outside the roundish aggregates, and eventually LCs were enclosed by round CD34^+^ cells (brown arrows in Fig. 4B3,B4,C1,C2) (Supplementary Fig. S3). The LC-CD34^+^ cell-aggregates did not adhere to the dish firmly, but instead were always staggering across it. It was noteworthy that when such aggregates came across each other, they sometimes touched each other and coalesced to make bigger aggregates (Fig. 4C1–C4). Before the coalescence, many thin cytoplasmic processes extending from CD34^+^ cells that were on the outside of each aggregate linked to such processes on the other aggregate (black arrows in Fig. 4C2–C4), and gradually shortened, finally resulting in coalescence into one aggregate in which the central part was occupied by LCs and the outer part was CD34^+^ cells (Fig. 4C1–C4) (Supplementary Video S3).

On the other hand, CD34^+^ cells which adhered to the bottom of the dish began to extend their cytoplasm about 3 h after inoculation and commenced to move at around 5-6 h following inoculation; some cells which assembled in some regions moved away from each other (yellow boxed areas in Fig. 4B1,B1’,B2,B2’) and some of them moved toward the LC-CD34^+^ cells-aggregates (purple arrows in Fig. 4B2’,B3’). Sometimes it was observed that the CD34^+^ cells extended thin cytoplasmic processes (filopodia) toward the LC-CD34^+^ cell-aggregates (a purple arrow in Fig. 4B3’) (Supplementary Fig. S3) and, after touching the aggregates with the process they entered into the aggregates (yellow arrows in Fig. 4B2’) (Supplementary Fig. S3; Supplementary Video S2). These phenomena by CD34^+^ cells were not observed toward aggregates which consisted of only CD34^+^ cells. CD34^+^ cells distributed around the CD34^+^ cell-aggregates never came toward the aggregates; instead, CD34^+^ cells within the aggregates tended to spread away from each other. When LCs and CD34^+^ cells were incubated with VCAM1 antibody and then mixed, LC-CD34^+^ cell-aggregates were also observed to form and gradually become roundish, with LCs enclosed by round CD34^+^ cells (Fig. 4D1–D4, like those treated with control antibodies (Fig. 4B1–B4). However, CD34^+^ cells that adhered to the dish initially showed less motion than those treated with control antibody. Some CD34^+^ cells which approached LC-CD34^+^ cell-aggregates sometimes failed to enter into the aggregates (white-boxed areas in Fig. 4D2,D2’,D3,D3’) (Supplementary Video S4). The number of CD34^+^ cells which entered the aggregates in the presence of VCAM1 antibody from 5 to 15 h following the inoculation (29 ± 10.7 cells in 3 experiments) was robustly lower (p < 0.01 by Multiple Comparison Procedure by Dunnett's test) than the number treated with control antibody (180 ± 22. 6 cells). When antibodies against integrins α4, α9 and β1 were added, LC-CD34^+^ cell-aggregates became roundish and LCs were finally enclosed by round CD34^+^ cells (Fig. 4E1–E4), as in the presence of control antibodies. CD34^+^ cells on the bottom of the dish were initially rounder in shape, and the percentage of the cells that adhered to the dish was significantly lower than that in the absence of the antibodies. But even round CD34^+^ cells that were situated very close to the LC-CD34^+^ cell-aggregates entered into the LC-CD34^+^ cell-aggregates while maintaining round shape and sometimes extending filopodia (white boxed area in Fig. 4E2,E3) (Supplementary Video S5). Also, CD34^+^ cells on the dish gradually extended their cytoplasm and some of them moved toward the aggregates to enter them. The number of CD34^+^ cells which entered the aggregates from 5 to 15 h following the inoculation (167.3 ± 32.4 cells) seemed not to be very different (p = 0.82) from that of cells treated with control antibody. These results indicate that VCAM1 plays important roles in the entrance of CD34^+^ cells into LC-CD34^+^ cell aggregates, but not in LC-LC adhesion or LC-CD34^+^ cell adhesion. On the other hand, α4, α9 and β1 integrins seem to be involved in adhesion to collagen and locomotive activity of CD34^+^ cells, but not in LC-LC adhesion or LC-CD34^+^ cell adhesion.

## Discussion

Two types of LCs, FLCs and ALCs, develop in rodents, and their origin, relationship, and regulatory mechanisms of development have been extensively investigated^16,17,26–29^. However, studies on the functional relationship of LCs to CD34^+^ cells are scarce. Here we showed some interesting differences in the behavior of FLCs and ALCs in relation to CD34^+^ cells in re-aggregate culture of dissociated testicular cells. The first is that the proliferative capacity of ALCs was significantly higher than that of FLCs. Given that CD34^+^ cells show high proliferative activity and similar proliferative pattern to that of ALCs during re-aggregate culture^12^, these results indicate that our in vitro re-aggregate culture system reflects in vivo situations well; the proliferative activity of FLCs is high during the late fetal stage, but declines after birth^26,27^. On the other hand, ALCs arise from stem cells in the neonatal testis, and the precursor cells of ALCs proliferate and differentiate to 3β-HSD-positive LC progenitors (PLCs)^13,28,29^. ALCs and mesenchymal cells increase in number linearly from birth through 90 days^29^. The PLCs further differentiate to immature Leydig cells (ILCs), and finally to mature Leydig cells (MLCs). As such, the ALCs with proliferative activities which we observed in re-aggregate culture are regarded as PLCs and/or ILCs. However, it remains to be clarified whether precursor cells differentiate into PLCs and ILCs, which differentiate into mature LCs in this re-aggregate culture. The second point, which seems to be one of the most intriguing results in the current study, is that ALCs were preferentially localized within CD34^+^ aggregates, whereas FLCs were not. Our immunofluorescence study showed that α4, α9, and β1 integrins as well as VCAM1 are expressed in both CD34^+^ cells and ALCs, but are barely expressed in FLCs. Our results accord well with previous reports in that the integrin β1 is expressed in PMCs and basement membrane^30–34^, but here we have made new findings that α4, α9 and β1 integrins are expressed in the CD34^+^ cells and ALCs, but not in FLCs. The present findings of VCAM1 expression in ALCs and CD34^+^ cells, but not in FLCs, accord well with its mRNA and protein expression pattern in interstitial cells and LCs in mouse testis^14,35–37^. Addition of function-blocking antibodies against α4, α9, and β1 integrins, and VCAM1 to the re-aggregate culture of testicular cells disturbed the reconstruction of ordered seminiferous tubule-like and interstitial-like structures, resulting in a smaller area of CD34^+^ cell-aggregates and a smaller number of LCs in addition to a rosette-like pattern of multi-layered Sertoli cells. One reason for the smaller number of CD34^+^ cells and LCs could be the decreased proliferative activities of CD34^+^ cells and ALCs, which were verified to have been caused by the addition of the antibodies. Cell adhesion by integrins to ECM leads to activation of Raf and mitogen activated protein (MAP) kinase kinase (MEK1), and translocation of the extracellular signal-regulated protein kinases 1 and 2 (Erk1/2) into the nucleus, finally resulting in expression of cyclin D1 and progression through the G1-S stage of the cell cycle^38^. Addition of soluble VCAM-1 promotes the proliferation of embryonic stem cell (ESC)-derived cardiomyocytes in monoculture^39^. TGF-β1 significantly increases expression of VCAM1 in human lung fibroblasts, and depletion of *VCAM-1* mRNA inhibits proliferation of fibroblasts by impairing cell cycle progression through depletion of phosphorylated p38, extracellular signal-regulated kinase ½ (ERK ½)^40^. Another possible reason is that the majority of the CD34^+^ cells could not join in the re-aggregate culture of testicular cells, because those antibodies inhibited the aggregation of CD34^+^ cells. This second possibility was also demonstrated to be true by the cell-to-cell adhesion assay of CD34^+^ cells, in that the antibodies against α4 and β1 integrins and VCAM1 inhibited the adhesion between purified CD34^+^ cells. Moreover, the assay of cell adhesion to recombinant VCAM1 demonstrated that CD34^+^ cells can bind to VCAM1. Loss of CD34^+^ cells in a re-aggregate during a re-aggregation process should have accompanied the loss of ALCs in the culture. VCAM1 was first identified as a cell adhesion glycoprotein molecule^41–43^ and is one of the ligands for α4β1 as well as α9β1 integrin^21^. In inflammation, Tumor Necrosis Factor (TNF)-α induces vascular endothelial cells to express VCAM1, which directly interacts with α4β1 integrin on leucocytes, followed by their firm adhesion to the endothelium, rolling and transendothelial migration^41,42^. Our current study indicates that CD34^+^ cells adhere to each other through interaction of α4β1 integrin with VCAM1.

Then, what is the answer to the key question of why ALCs were re-localized within CD34^+^ cell-aggregates in the re-aggregate culture of testicular cells? Observations by live cell imaging showed some intriguing behaviors of ALCs and CD34^+^ cells. Firstly, when CD34^+^ cells were mixed with ALC-aggregates, they easily bound the ALCs, indicating strong adhesiveness to ALCs. Moreover, when two CD34^+^ cell-ALC-aggregates happened to meet, they coalesced into one aggregate and the CD34^+^ cells came to be localized in the outer part of the aggregate, while the ALCs came to be in the inner part. This phenomenon can be regarded as sorting out of CD34^+^ cells and ALCs. This may be one reason why ALCs were localized within the CD34^+^ cell-aggregates. Secondly, some CD34^+^ cells that adhered to the bottom of the dish were seen to change in shape to become elongated in a front-back direction, and to move toward the ALC-CD34^+^ cell-aggregates. Sometimes it was clearly observed that CD34^+^ cells extended thin filopodia toward the aggregate, and after touching it with the filopodia they plunged into the aggregate. These phenomena were not observed toward aggregates which consisted of only CD34^+^ cells. Hence it seems reasonable to conclude that CD34^+^ cells move toward ALCs of the ALC-CD34^+^ cell-aggregates, presumably by chemotaxis or haptotaxis. Addition of VCAM1 antibody to the culture reduced the frequency of CD34^+^ cells which approached and entered into the ALC-CD34^+^ cell-aggregates, indicating a possibility that VCAM1 acts as an attractant for CD34^+^ cells. VCAM1 is involved in the development of some malignant tumors, and overexpression of VCAM1 facilitates metastasis by promoting epithelial-mesenchymal transformation and transendothelial migration by enhancing pseudopodia formation^43^. Some types of cancer cells, such as pancreatic cancer cells, secrete VCAM1, which attracts macrophages to the tumor environment^44^. It may be reasonable to assume that filopodia that are frequently observed in CD34^+^ cells and extended toward ALC-CD34^+^ cell-aggregates respond to the haptotic gradient of VCAM1^45^. α4β1 integrin plays an important role in adhesion of leukocytes to endothelial cells and their transmigration through binding to VCAM1^41–43^. Hence, we propose, based on the current findings, a possibility that ALCs release VCAM1 as an attractant to which CD34^+^ cells respond, and thus CD34^+^ cells come to enclose them. It is possible, however, that VCAM1 antibody affected motion of CD34^+^ cells which express VCAM1. Antibodies against α4, α9 and β1 integrins did not show an apparent inhibitory effect on the frequency of entry of CD34^+^ cells into the ALC-CD34^+^ cell-aggregates, indicating a possibility that other integrins are involved in this phenomenon. It remains to be determined whether ALCs release VCAM1, and whether CD34 + cells move toward VCAM1 or not.

Then, how can we extrapolate the current in vitro results to testicular development in vivo? Candelaria et al*.* recently reported that VCAM1 mRNA and protein expression is selectively and highly elevated in theca cells and interstitial cells when mouse ovary is exposed to high androgen levels^36^. It is possible that VCAM1 is also related to androgen levels in testes and thus to functions in development of spermatogenesis. On the other hand, in the course of the differentiation as well as in regeneration of ALCs in ethylene dimethane sulphonate-treated rats, mesenchymal cells (CD34^+^ cells) arise at a peritubular position as their precursor cells, and show increased cell growth, while LCs are damaged^46,47^. The precursor cells move toward the central interstitium to make clusters, while differentiating to mature ALCs^17,29,46^. Eventually, interstitial cells enclose elongated Leydig cells within the thin cytoplasmic extensions^46–49^. It is conceivable that CD34^+^ cells regulate the proliferation and differentiation of ALCs, and that they encase differentiating ALCs and convey or help them to move toward the final position along the basement membrane.

Our current study showed that re-aggregate culture of the dissociated cells from 10-dpp testes resulted in reconstruction of testicular structures in which aggregates of interstitial cells including ALCs are surrounded by a layer of Sertoli cells, while we previously reported that seminiferous tubule-like structures are surrounded by interstitial cells when culture is started from 6-dpp testicular cells^11^. The inverted structures of seminiferous tubule-like structures and interstitial tissues formed from 10-dpp testicular cells may be due to the differences in the stage-dependent properties of the testicular cells. What determines ‘which surrounds which’ remains to be clarified in future. Although we can’t exclude a possibility that our in vitro re-aggregate culture system does not recapitulate in vivo situations correctly, various new findings obtained in the current study will provide deep insights into the regulatory mechanisms whereby cell–cell interactions and molecular networks operate to form and maintain the elaborate testicular structure in vivo.

## Methods

### Ethics statement

The study was performed using protocols approved by the Animal Care and Use Committee of Kumamoto Health Science University (Approval Number: 18-14). All experiments were performed in accordance with the relevant guidelines and regulations, and in compliance with the ARRIVE guidelines (https://arriveguidelines.org).

### Mice

Two lines of transgenic mice, *mFLE-EGFP*^23^, and *Ad4BP-BAC-EGFP* mice^25^ were established as described previously. In the former line, EGFP is expressed in FLCs under the control of an FLC specific enhancer of *Ad4BP* gene (*Nr5a1*). For the latter line, a BAC clone containing a whole *Ad4BP* gene locus was used. Therefore, EGFP is expressed in both FLCs and ALCs in the mice. These transgenic mice are maintained by mating with C57BL/6J. In addition, C57BL/6J were used as wild type mice. These mice were maintained on a 12-h day/12-h night schedule at constant temperature and humidity in the Center for Animal Resources at Kumamoto Health Science University according to protocols for the animal experiments approved by the Institutional Animal Care and Use Committees.

### Re-aggregate culture of dissociated testicular cells

Testes from 3 to 8 mice at 9- or 10-dpp, which contain comparable numbers of FLCs and ALCs, were used per experiment. Dissociation of the testes, re-aggregation, and culture in a 3-D system were performed as previously reported^12^ with some modifications. Briefly, after the tunica albuginea was removed, whole testes were diced, and treated with 0.08% collagenase (type IV, Worthington) and 0.1% hyaluronidase (type I-S, Sigma) for 1 h at room temperature (RT), and then with 0.01% DNase I (Sigma) for the final 5 min, followed by thorough dispersal by pipetting and filtering through a nylon mesh filter (70 μm, Milteny 130-098-462). After washing in RPMI-1640 medium (Wako, Japan), cell suspension was re-aggregated on a shaker (60 rotations/min, TAITEC, Japan) for 1 h at RT. The cell suspension was divided into 10–20 siliconized tubes (0.5 ml) (Assist, Japan) so that one tube contained 7–8 × 10^5^ cells, followed by centrifugation at 230×*g* for 5 min. Each cell pellet was sucked up by a pipetman with a tip containing 70 μl collagen (Cellmatrix Type I-P, Nitta Gelatin, Japan, final concentration 2.4 mg/ml) in the medium, and put on a Nuclepore filter (Whatman, USA). After the collagen hardened, cell pellet on the filter was cultured in a medium containing 10% KSR (Gibco, USA) at 34 °C in a dish (Falcon 351008) in a humidified incubator (APM-30D, ASTEC, Japan) with an atmosphere of 5% CO_2_ and 50% O_2_. Cell suspensions were incubated with function-blocking antibodies (Supplementary Table S1) for 15 min on ice before rotation. Areas which CD34^+^ cell-aggregates occupied within a whole area of a section of a testicular re-aggregate were delineated using a tool installed in a fluorescence microscope (BX61VS-ASW, Olympus), and ratios of areas which CD34^+^ cell-aggregates occupied to the total area of a re-aggregate were obtained as an average percentage in 3 sections of a re-aggregate. The average values (means ± S.E.M) were obtained from 3 experiments.

Proliferative activities of CD34^+^ cells and LCs were obtained by incubation with 20 μM 5-bromo-2-deoxy-uridine (BrdU, Sigma) for 16 h during re-aggregate cultures and the sections were processed for immunohistochemistry with a kit according to the manufacturer’s instructions (Amersham, England). The number of cells positive for BrdU and CD34, GFP or HSD3β1, and the total number of CD34-, GFP- and HSD3β1-positive cells were counted in 3 photographed areas selected at random each in 3 sections. These 3 sections consisted of every eighth sections (40 μm apart) in a sample. The proliferative activities of CD34^+^ cells, FLCs, and LCs (ALCs + FLCs) were obtained as a percentage of the cells that were positive for BrdU and CD34, GFP or HSD3β1, relative to the total number of CD34-, GFP- and HSD3β1-positive cells, respectively. The average values (means ± S.E.M.) from 3 independent experiments were calculated.

### Immunofluorescence staining

Fourteen-dpp testes of wild type and *mFLE-EGFP* mice were frozen at – 20 °C, and embedded in OCT compound (Sakura Finetek, Japan). Cryosections (7 μm) were fixed in methanol and acetone (the ratio was 1:1, but when stained with anti-GFP antibody, the ratio was 1:3) for 7 min at − 20 °C. The sections were blocked by 5% serum derived from the same animal as that of the secondary antibody in TNB blocking buffer (PerkinElmer FP1020) for 1 h, followed by staining with the first antibody at 4 °C overnight. After washing with PBS, the sections were incubated with the secondary antibody for 1.5 h at RT. *mFLE-EGFP* mouse testes were used for co-staining with GFP antibody and antibodies against integrin α4, α9 (Fujifilm Wako) or VCAM1. But when stained with integrin β1 antibody, GFP antibody was not used (because both antibodies are derived from rabbit); GFP fluoresced spontaneously. 4′6′-diamidino-2-phenylindole (DAPI) (Sigma) was used for nuclear staining.

Cultured re-aggregates were fixed in 4% paraformaldehyde (PFA) overnight and embedded in paraffin. Sections (5 μm) were steamed in histofine solution, pH9 (Nichirei Biosciences 415211) for 15 min to unmask antigen epitopes. The sections were washed in PBS containing 0.1% Tween 20 (PBST), transferred to blocking solution mentioned above containing 0.3% Triton X-100 in TNB for 1 h, and stained with the first antibody and then with the secondary antibody as described above. Microscopic images were obtained using a CCD camera (DP72, Olympus, Tokyo) mounted on a fluorescence microscope (BX61VS-ASW, Olympus). The list of antibodies used is shown in Supplementary Table S1.

### Cell sorting

CD34^+^ cells were collected from 10- to 12-dpp testes from 3 to 8 wild type or *mFLE-EGFP* transgenic mice by fluorescence-activated cell sorting. Dissociated testicular cells were filtered through 30 μm nylon mesh, blocked with normal mouse serum, and stained with Brilliant Violet 421™ (BV421) anti-mouse CD34 antibody (BL 119321) in cell staining buffer (CSB) {Hanks balanced salt solution (Wako) containing 1% BSA (Sigma‐Aldrich, MO) and 5% FBS (Thermo)}. After washing with CSB, cell sorting was performed using a cell sorter SH800 (Sony, Japan). (Acquired data were analyzed using FlowJo software (FlowJo LLC, Ashland, OR, http://www.flowjo.com). Unstained, single stained, and isotype matched controls were used for gating strategies. EGFP-positive LCs including ALCs and FLCs were collected from 10- to 16-dpp testes from 1 to 4 *Ad4BP-BAC-EGFP* mice by separating GFP^+^ cells from CD34^+^ GFP^+^ cells (Supplementary Fig. S2).

### Cell–cell adhesion assay of testicular cells and CD34^+^ cells

To examine the extent of aggregation of dissociated testicular cells and CD34^+^ cells, 2 × 10^5^ cells in 0.5 ml of RPMI medium were preincubated with function-blocking antibodies as described above. The cells were put into each well of a 24-well flat bottom plate (Violamo, VTC-P24) that had been coated with 0.1% BSA in RPMI medium overnight, and rotated on a shaker (70 rpm) for 1 h at RT. Then 0.5 ml of PFA was added to each well, gently mixed, and the number of particles was counted in triplicate for each sample using a hemocytometer (Fuchs Rosenthal). Extent of aggregation was expressed as 1 − Nt/No, where No is the number of particles (almost single cells) at starting time, and Nt is that after 1 h of aggregation. Average values ± S.E.M. for 3 experiments were obtained.

### Cell-adhesion assay of CD34^+^ cells to VCAM1

Flat bottom Maxisorp 96-well immuno plates (442404 Thermo Fisher Scientific, Denmark) were coated with 0.1% BSA in RPMI (negative control) or 10 μg/ml recombinant mouse VCAM1/Fc (RSD-0956-43 R&D Systems, MN) overnight at 4˚C. After washing with PBS, coated wells were blocked with 0.5% BSA in PBS for 1 h at RT. Plates were then washed once with PBS, followed by application of 7.5 × 10^4^ cells in 200 μl of 0.25% BSA in RPMI medium and incubated for 1 h at 34 °C. Cells were preincubated with function-blocking antibodies as described above. After washing with PBS once, 50 μl of 0.5% crystal violet (Katayama Chemical, Japan) in 20% methanol was added to each well for 30 min at RT, and then wells were washed with tap water more than 3 times. After adding 100 μl of 20% acetic acid to each well, absorbance was measured at 595 nm using an iMark microplate reader (Bio-Rad). Average values ± S.E.M. for 3 experiments (duplicate for each) were obtained.

### Live cell imaging of LCs and CD34^+^ cells

CD34^+^ cells and GFP-positive LCs (ALCs and FLCs) were isolated by cell sorting as described above. Each fraction was treated with control antibodies (hamster and rat), VCAM1 antibody, or mixture of α4, α9 and β1 integrin antibodies for 30 min on ice, the LCs fraction was warmed at 34 °C for 30 min, and then LCs only (1 × 10^5^ cells) or LCs plus CD34^+^ cells (2 × 10^5^ cells) in 150 μl in RPMI medium containing 10% KSR were put on glass base dishes (IWAKI, Japan) directly or on round coverslips (C1100, Matsunami, Japan), both of which had been coated with collagen (Type I-C, Nitta Gelatin). Three glass base dishes with or without round coverslips were placed in a humidified chamber of confocal microscope (CV1000, Yokogawa, Japan). Time-lapse imaging was performed over 1–3 days period with a ×10 objective lens at 34 °C with 50% O_2_ and 5% CO_2_ using a 488 nm laser. Confocal optical Z-sections were collected every 2 μm throughout 20 μm at 15-min intervals. All time-lapse images presented in this report show slice planes except LCs only in which case maximum-intensity projections (MIPs) were taken.

### Statistics

Shapiro–Wilk normality test was performed for all groups. Student’s *t*-test was used to compare two groups containing data which follow a normal distribution. Mann–Whitney U test was used to compare two groups containing data which did not follow a normal distribution. When 3 groups were compared, all data were confirmed to fit a normal distribution, then we adopted one-way ANOVA with Dunnett's post-hoc multiple comparison procedure. *P* values less than 0.05 were considered statistically significant. Summary of the results are shown as Supplementary Table S2.

## Supplementary Information

Supplementary Information 1.

Supplementary Information 2.

Supplementary Video 1.

Supplementary Video 2.

Supplementary Video 3.

Supplementary Video 4.

Supplementary Video 5.
