# Supplementary material for: VCAM1-α4β1 integrin interaction mediates interstitial tissue reconstruction in 3-D re-aggregate culture of dissociated prepubertal mouse testicular cells
Source: Sci Rep. 2021 Sep 15;11:18332. doi: 10.1038/s41598-021-97729-y (PMC8443749; doi:10.1038/s41598-021-97729-y)
Supplement: Supplementary file 1 — Supplementary Information 1. [file 41598_2021_97729_MOESM1_ESM.pdf]

VCAM1-$\alpha4\beta1$ integrin interaction mediates interstitial tissue reconstruction in 3-D re-aggregate culture of dissociated prepubertal mouse testicular cells

Kazuko Abe[1], Shigeyuki Kon[2], Hiroki Kameyama[1], JiDong Zhang[3], Ken-ichirou Morohashi[4], Kenji Shimamura[5], Shin-ichi Abe[1*]

[1]Faculty of Health Science, Kumamoto Health Science University, Japan, [2]Department of Molecular Immunology, Faculty of Pharmaceutical Sciences, Fukuyama University, Fukuyama, Hiroshima, Japan, [3]School of Basic Medical Sciences, ZunYi Medical University, Zunyi, Guizhou Province, China, [4]Department of Molecular Biology, Graduate School of Medical Sciences, Kyushu University, Fukuoka, Japan, [5]Institute of Molecular Embryology and Genetics, Kumamoto University, Kumamoto, Japan

Supplementary Methods

Calculation of the percentage of the number of ALCs which were localized within $CD34^{+}$ cell-reaggregates

1) Percentage of the number (No.) of ALCs which were localized within $CD34^{+}$ cell-reaggregates among all the ALCs in 3 sections on day 7 of each culture was obtained by double immunostaining of CD34 and HSD3β1, or GFP, as well as of HSD3β1 and GFP.
2) The ratio of the No. of FLCs (GFP^{+} cells) localized within $CD34^{+}$ cell-reaggregates to the No. of all the GFP^{+} cells counted was designated as 'X'.
3) The ratio of the No. of ALCs (No. of $HSD3\beta1^{+}$ cells – No. of FLCs) to the No. of $HSD3\beta1^{+}$ cells, was designated as 'Y'.
4) The ratio of the No. of FLCs to that of $HSD3\beta1^{+}$ cells was designated as 'Z'.
5) Then, the percentage of ALCs localized within $CD34^{+}$ cell-reaggregates among all the ALCs counted on day 7 was obtained as {No. of $HSD3\beta1^{+}$ cells localized within $CD34^{+}$ cell-reaggregates – (No. of $HSD3\beta1^{+}$ cells in sections x 'Z' x 'X')} x 100 / (total No. of $HSD3\beta1^{+}$ cells in sections x 'Y').
6) An average ± S.E.M. was obtained for 3 experiments.

Calculation of the proliferative activity of ALCs

1) Proliferative activity of ALCs was obtained by double immunostaining of BrdU and HSD3β1, or GFP, as well as of HSD3β1 and GFP, in 3 sections for each day (1, 3, 5, 7d) of each culture.
2) The ratio of $BrdU^+$ FLCs to all the FLCs (GFP^+ cells) in some randomly selected areas of the sections on each day of culture was designated as 'X'.
3) The ratio of the No. of ALCs (No. of $HSD3\beta1^+$ cells – No. of GFP^+ cells) to the No. of $HSD3\beta1^+$ cells was designated as 'Y'.
4) The ratio of the No. of FLCs to the No. of $HSD3\beta1^+$ cells was designated as 'Z'.
5) Then, the proliferative activity of ALCs (percentage of the $BrdU^+$ALCs among all the ALCs counted) on one day of culture was obtained as (No. of $BrdU^+HSD3\beta1^+$ cells – No. of $HSD3\beta1^+$ cells x 'Z' x 'X') x 100/ (No. of $HSD3\beta1^+$ cells x 'Y').
6) Average percentage ± S.E.M. was obtained for 3 experiments.

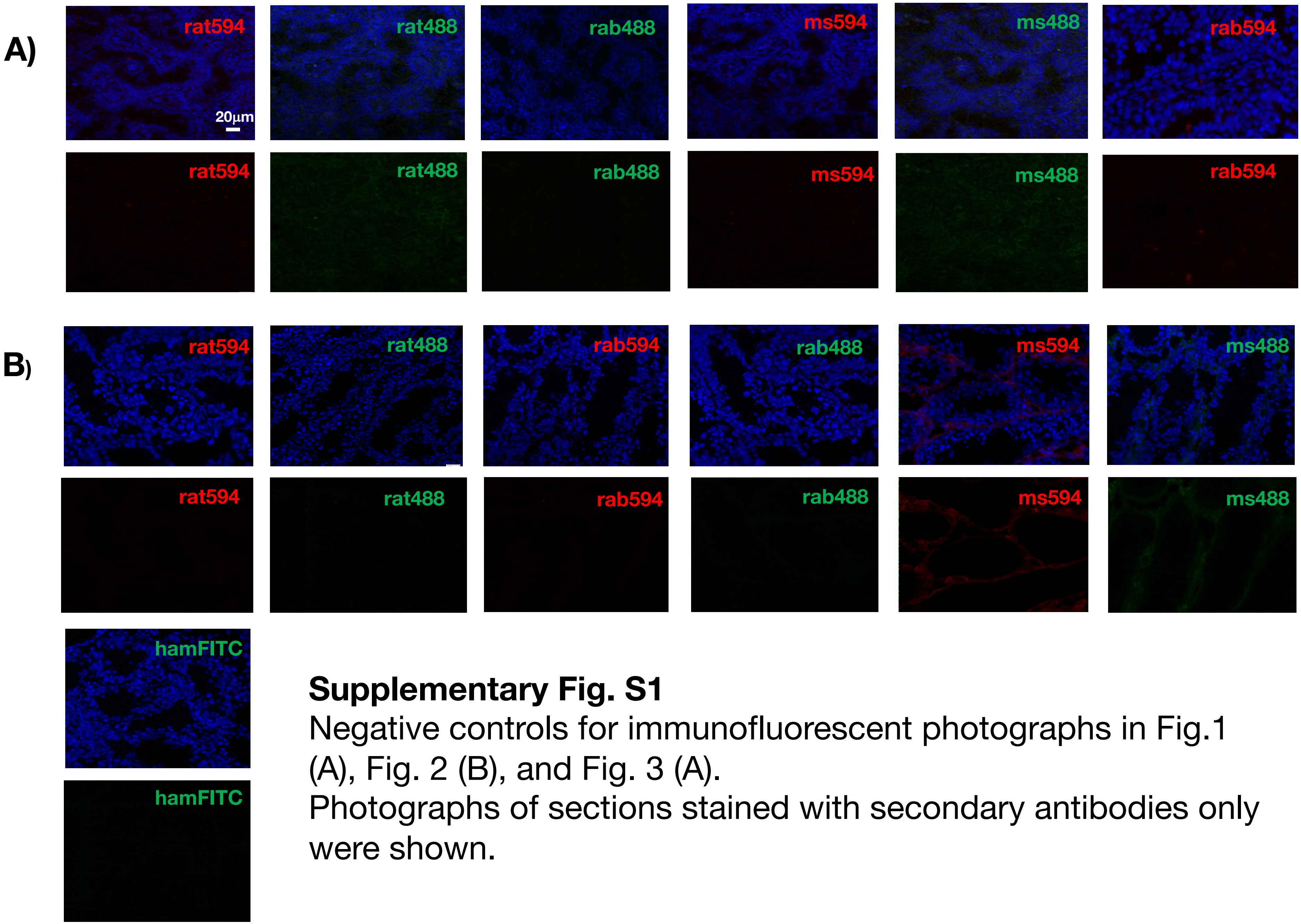

Supplementary Fig. S1

Negative controls for immunofluorescent photographs in Fig.1 (A), Fig. 2 (B), and Fig. 3 (A).

Photographs of sections stained with secondary antibodies only were shown.

A) Wild type B) *mFLE-EGFP* C) *Ad4BP-BAC-EGFP*

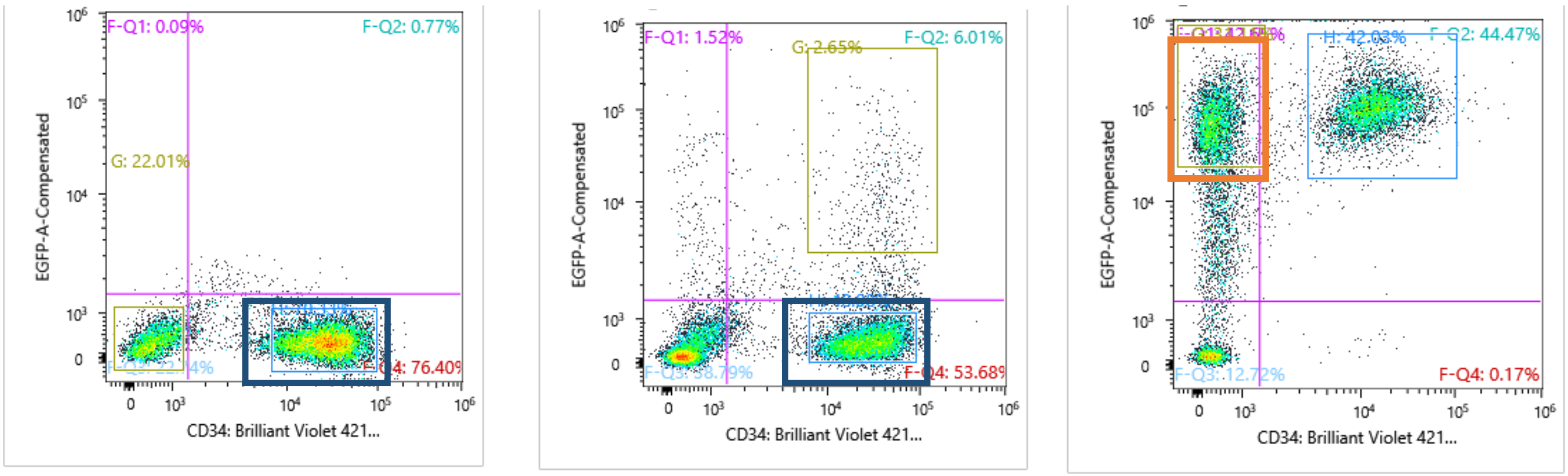

Supplementary Fig. S2

Fractions surrounded by blue boxes in (A) and (B) were sorted as $CD34^{+}$ cells from wild type and *mFLE-EGFP* transgenic mice, respectively.

Fractions surrounded by an orange box in (C) were sorted as LCs (ALCs and FLCs) from *Ad4BP-BAC-EGFP* transgenic mice.

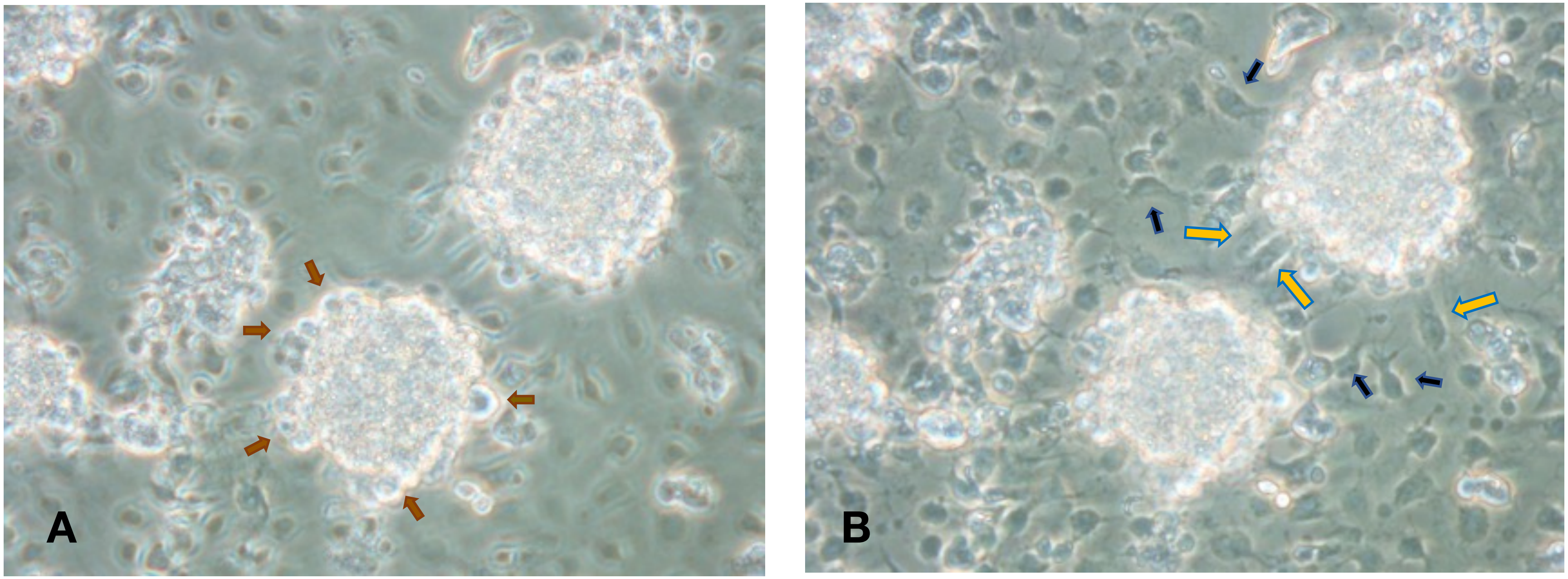

Supplementary Fig. S3

Phase contrast microscopy shows LC-CD34^{+} cell aggregates and CD34^{+} cells that attached on dishes and extended around the aggregates.

A) Focus is on some CD34^{+} cells attached around LC-aggregates (brown arrows).

B) Focus is on CD34^{+} cells which attached and extended on the dish.

Blue arrows indicate CD34^{+} cells which are approaching LC-CD34^{+} cell aggregates by extending thin filopodia. Orange arrows show CD34^{+} cells which are just plunging into LC-CD34^{+} cell-aggregates.
