# Supplementary material for: VCAM1-α4β1 integrin interaction mediates interstitial tissue reconstruction in 3-D re-aggregate culture of dissociated prepubertal mouse testicular cells
Source: Sci Rep. 2021 Sep 15;11:18332. doi: 10.1038/s41598-021-97729-y (PMC8443749; doi:10.1038/s41598-021-97729-y)
Supplement: Supplementary file 2 — Supplementary Information 2. [file 41598_2021_97729_MOESM2_ESM.pdf]

Supplementary Table S1. List of antibodies used

Antibodies used for immunofluorescence	animals	Vendor	Catalog no.	Working dilution
anti-human GATA-4	mouse	Santa Cruz	sc-25310	1:100
anti-mouse CD34	rat	BioLegend	119301	1:200
anti-GFP	rabbit	GeneTex	GTX113617	1:500
anti-mouse Hsd3β	rabbit	TransGenic	KO607	1:100
anti-integrin α4	rat	ATCC	PS/2	1:50
anti-CD34 (EP373Y)	rabbit	Abcam	ab81289	1:200
anti-α-SMA	mouse	Sigma-Aldrich	A 2547	1:10000
anti- α9 integrin	hamster	*	12C4'58	1:200
anti-integrin α9, monoclonal antibody(YA4), chicken/mouse IgG1・κ chimera	chicken/ mouse chimera	FujiFilm Wako	010-28061	1:500
anti-integrin β1 / CD29	rabbit	GeneTex	GTX128839	1:200
anti-HSD3β1	rat	**	2A8E3	1:100
anti-CD106(VCAM-1)	rat	ThermoFisher	16-1061-82	1:50
anti-rabbit IgG H&L (Alexa Fluor® 488)	donkey	Abcam	ab150073	1:500
anti-rabbit IgG H&L (Alexa Fluor® 594)	donkey	Abcam	ab150076	1:500
anti-rat IgG H&L (Alexa Fluor 488)	donkey	Invitrogen	A21208	1:500
anti-rat IgG H&L (Alexa Fluor® 594)	donkey	Abcam	ab150156	1:500
anti-mouse IgG H&L (Alexa Fluor® 488)	donkey	Abcam	ab150105	1:500
anti-mouse IgG H&L (Alexa Fluor® 594)	donkey	Abcam	ab150108	1:500
Fluorescein (FITC) AffiniPure Goat Anti-Syrian Hamster IgG (H+L)	goat	Jackson Immuno Research	107-095-142	1:200

Function-blocking antibodies				
IgG1 isotype control	rat	BL	400432	20 μg/m
IgG isotype control	Syrian hamster	BL	402034	20 μg/ml
anti-rat CD29 (β1 integrin)	hamster	BD	555002	10 μg/ml
anti-mouse CD106 (VCAM1)	rat	Invitrogen	16-1061-85	10 μg/ml,
anti-α4 integrin	rat	ATCC	PS/2	20 μg/ml
anti-α9 integrin	Syrian hamster	*	55A2C	20 μg/ml

*anti-α9 integrin was supplied by Kidswell Bio Corporation (Tokyo, Japan).

Kanayama et al., (2009) J. Immunol. 182(12):8015-25. doi: 10.4049/jimmunol.0900725.

**Yokoyama et al., (2019) Biochem. Biophys. Res. Comm. (2019) 511(4):916-920.
doi: 10.1016/j.bbrc.2019.02.100.

Supplementary Table S2. Statistical Analysis

Figure	groups and pairs	Shapiro-Wilk test			t-test			Mann-Whitney U-test	
		W	p	p <	t	p	p <	w	p
Fig.1H									
	ALC	0.97	0.65						
	FLC	0.99	0.77						
	ALC vs FLC				49.23	1.02E-06	<0.001 ***		
Fig. 1I									
	FLC1d	0.81	0.15						
	FLC3d	0.95	0.56						
	FLC5d	0.98	0.75						
	FLC7d	0.98	0.77						
	ALC1d	0.96	0.62						
	ALC3d	0.81	0.14						
	ALC5d	0.91	0.42						
	ALC7d	0.89	0.35						
	FLC1d vs ALC1d				2.78	0.04957	<0.05*		
	FLC3d vs ALC3d				6.47	0.002943	<0.01**		
	FLC5d vs ALC5d				6.82	0.002416	<0.01**		
	FLC7d vs ALC7d				14.37	0.0001363	<0.001***		
Fig. 3G									
	CtrlAb(ham)01	0.76	0.03						
	α9ITGAb	0.93	0.51						
	CtrlAb(ham)02	0.96	0.61						
	β1ITGAb	0.91	0.41						
	CtrlAb(rat)01	0.83	0.2						
	α4ITGAb	0.93	0.48						
	CtrlAb(rat)02	0.98	0.7						
	VCAM1Ab	1	0.93						
	CtrlAb(ham)01 vs α9ITGAb							9	0.1
	CtrlAb(ham)02 vs β1ITGAb				6.06	0.003747	<0.01**		
	CtrlAb(rat)01 vs α4ITGAb				5	0.007516	<0.01**		
	CtrlAb(rat)02 vs VCAM1Ab				8.11	0.001256	<0.01**		
Fig. 3H									
	CtrlAb(ham)01	0.82	0.17						
	α9ITGAb	0.94	0.52						
	CtrlAb(ham)02	0.98	0.71						
	β1ITGAb	not applicable because all the values are same							
	CtrlAb(rat)01	1	0.92						
	α4ITGAb	1	0.97						
	CtrlAb(rat)02	0.99	0.86						
	VCAM1Ab	0.75	<2.2e-16	<0.001***					

CtrlAb(ham)01 vs α9ITGAb	5.75	0.004547	<0.01 **		
CtrlAb(ham)02 vs β1ITGAb				9	0.064
CtrlAb(rat)01 vs α4ITGAb	3.87	0.01799	<0.05 *		
CtrlAb(rat)02 vs VCAM1Ab				9	0.077

Fig. 3I

CtrlAb(ham)01	0.8	0.12	
α9ITGAb	0.86	0.26	
CtrlAb(ham)02	0.92	0.46	
β1ITGAb	0.88	0.33	
CtrlAb(rat)01	0.81	0.14	
α4ITGAb	1	0.91	
CtrlAb(rat)02	1	0.88	
VCAM1Ab	0.95	0.55	

CtrlAb(ham)01 vs α9ITGAb	4.69	0.009412	<0.01 **		
CtrlAb(ham)02 vs β1ITGAb	8.38	0.001109	<0.01 **		
CtrlAb(rat)01 vs α4ITGAb	6.23	0.003379	<0.01 **		
CtrlAb(rat)02 vs VCAM1Ab	8.45	0.001072	<0.01 **		

Fig. 3J

CtrlAb(ham)01	0.87	0.31	
α9ITGAb	0.92	0.44	
CtrlAb(ham)02	0.77	0.03403	<0.05 *
β1ITGAb	0.83	0.18	
CtrlAb(rat)01	0.98	0.71	
α4ITGAb	0.92	0.44	
CtrlAb(rat)02	0.95	0.57	
VCAM1Ab	0.95	0.58	

CtrlAb(ham)01 vs α9ITGAb	1.14	0.3194			
CtrlAb(ham)02 vs β1ITGAb				4	1
CtrlAb(rat)01 vs α4ITGAb	1.57	0.1918			
CtrlAb(rat)02 vs VCAM1Ab	0.84	0.4492			

Fig. 3K

CtrlAb(rat)01	0.86	0.26	
α4ITGAb	1	0.9	
CtrlAb(rat)02	0.99	0.85	
VCAM1Ab	1	0.89	
CtrlAb(ham)01	0.81	0.14	
β1ITGAb	0.83	0.18	
CtrlAb(ham)02	0.76	0.0075	<0.01 **
α9ITGAb	0.86	0.27	

CtrlAb(rat)01 vs α4ITGAb	5.78	0.004455	<0.01 **		
CtrlAb(rat)02 vs VCAM1Ab	5.52	0.005243	<0.01 **		
CtrlAb(ham)01 vs β1ITGAb	4.95	0.007786	<0.01 **		
CtrlAb(ham)02 vs α9ITGAb				2	0.4

	Shapiro-Wilk test			t-test			Mann-Whitney U-test	
	W	p	p <	t	p	p <	w	p
**Fig. 3L**								
CtrlAb(rat)01	0.96	0.6						
α4ITGAb	0.77	0.03404	<0.05 *					
CtrlAb(rat)02	0.86	0.27						
VCAM1Ab	0.86	0.26						
CtrlAb(ham)01	0.86	0.27						
β1ITGAb	0.87	0.29						
α9ITGAb	0.78	0.07						
CtrlAb(rat)01 vs α4ITGAb							9	0.1
CtrlAb(rat)02 vs VCAM1Ab				8.11	0.001259	<0.01 **		

	One-way Anova			Multiple Comparison Procedure by Dunnett's test		
	F	p	p <	t	p	p <
CtrlAb(ham)01 vs β1ITGAb vs α9ITGAb	10.4	0.0112	<0.01 **			
CtrlAb(ham)01 vs β1ITGAb				4.05	0	<0.05 *
CtrlAb(ham)01 vs α9ITGAb				0.22	1	

	W	p
**Fig. 3M**		
CtrlAb(rat)	0.88	0.32
α4ITGAb	0.88	0.32
CtrlAb(ham)	1	0.96
β1ITGAb	0.82	0.17
α9ITGAb	0.93	0.48

	t-test		
	t	p	p <
CtrlAb(rat)01 vs α4ITGAb	13	2.00E-04	p<0.001 ***

	One-way Anova			Multiple Comparison Procedure by Dunnett's test		
	F	p	p <	t	p	p <
CtrlAb(ham) vs β1ITGAb vs α9ITGAb	14.66	0.0049	<0.01 **			
CtrlAb(ham)01 vs β1ITGAb				5.35	0	<0.01 **
CtrlAb(ham)01 vs α9ITGAb				1.95	0.2	
